# Supplementary material for: Fast inhibition slows and desynchronizes mouse auditory efferent neuron activity
Source: bioRxiv. 2024 Jan 23:2023.12.21.572886. Preprint. [Version 2] doi: 10.1101/2023.12.21.572886 (PMC10836066; doi:10.1101/2023.12.21.572886)
Supplement: Supplement 1 [file NIHPP2023.12.21.572886v2-supplement-1.pdf]

bioRxiv preprint doi: https://doi.org/10.1101/2023.12.21.572886; this version posted January 23, 2024. The copyright holder for this preprint (which was not certified by peer review) is the author/funder. This article is a US Government work. It is not subject to copyright under 17 USC 105 and is also made available for use under a CC0 license.

Supplementary Methods

RESOURCE AVAILABILITY

Materials availability

This study did not generate new unique materials.

Data and code availability

DATA

Electrophysiology and imaging data will be deposited and publicly available by the date of publication.

CODE

All original code will be deposited and will be publicly available as of the date of publication.

ANALYSIS TOOLS

Any additional information required to reanalyze the data reported in this paper is available from the lead contact upon request.

EXPERIMENTAL MODEL DETAILS

Ethical approval and animal housing

Animal procedures followed National Institutes of Health guidelines, as approved by the National Institute of Neurological Disorders and Stroke/National Institute on Deafness and Other Communication Disorders Animal Care and Use Committee. Pre-weaned mice postnatal age 13-21 (P13-P21) were used for experiments and were housed with parents and littermates before use. Mice were housed in the NIDCD animal facility with a 12:12 light/dark cycle where food and water were provided *ad libitum*. Mice of both sexes were used for experiments. For consideration of sex as a biological factor, postsynaptic currents (PSCs) recorded during stimulation of the ventral acoustic stria ('midline stimulation'; MdL-stimulation) were analyzed and compared between the sexes (see below for complete methods). No significant differences

bioRxiv preprint doi: https://doi.org/10.1101/2023.12.21.572886; this version posted January 23, 2024. The copyright holder for this preprint (which was not certified by peer review) is the author/funder. This article is a US Government work. It is not subject to copyright under 17 USC 105 and is also made available for use under a CC0 license.

were found for excitatory or inhibitory PSCs using the metrics of onset latency, onset jitter, rise time, decay tau, amplitude and probability. Datasets were therefore pooled.

Median ± MAD		Onset Latency (ms)	Onset Jitter (ms)	Rise Time (ms)	Decay Tau (ms)	Amplitude (nA)	Probability
Male	EPSCs (10)	2.11 ± 0.43	0.19 ± 0.08	0.61 ± 0.28	2.44 ± 0.50	-0.05 ± 0.02	0.48 ± 0.21
Female	EPSCs (11)	2.02 ± 0.67	0.41 ± 0.15	0.73 ± 0.20	2.23 ± 0.55	-0.04 ± 0.02	0.52 ± 0.31
Male	IPSCs (8)	5.01 ± 1.98	0.53 ± 0.08	1.26 ± 0.59	5.79 ± 2.85	-0.07 ± 0.02	0.39 ± 0.11
Female	IPSCs (10)	5.30 ± 1.26	0.44 ± 0.20	1.40 ± 0.32	3.34 ± 0.48	-0.05 ± 0.02	0.25 ± 0.16

Table M1: Values for metrics analyzed for PSCs evoked with midline (MdL) stimulation for comparison of sex as a biological factor. n # for each group in parentheses represents # of peaks (clusters) analyzed. Comparisons made between sexes showed no significant differences with Mann-Whitney U Test.

ChAT-IRES-Cre x tdTomato mouse line

ChAT-IRES-Cre transgenic mice on either a C57BL/6J (RRID:IMSR_JAX:028861) or a C57BL/6N (RRID:IMSR_JAX:018957) background strain were crossed with tdTomato reporter mice (Ai14, Cre reporter allele inserted into Rosa 26 locus; RRID:IMSR_JAX:007914) to yield offspring heterozygous for each allele for experiments. These mice were used to target MOC neurons for patch-clamp recordings as previously described (Torres Cadenas et al., 2020).

Atoh7/Math5 Cre x GCaMP6f mouse line

Atoh7/Math5 Cre mice (Yang et al., 2003, RRID:MGI:3717726) were crossed with GCaMP6f Ai95(RCL-GCaMP6f, RRID:IMSR_JAX:028865) mice for use in calcium imaging experiments of cochlear nucleus bushy cells.

Brain slice preparation

Mice were killed by carbon dioxide inhalation at a rate of 20-30% of chamber volume per minute, then decapitated. The brain was removed in cold artificial cerebrospinal fluid (aCSF) containing the following (in mM): 124 NaCl, 1.2 $CaCl_2$, 1.3 $MgSO_4$, 5 KCl, 26 $NaHCO_3$, 1.25 KH_2PO_4, and 10 dextrose; 1 mM kynurenic acid was included during slice preparation. The pH was equal to 7.4 when bubbled with 95% O_2/5% CO_2. In experiments in which mini-post-synaptic potentials (mPSPs) were recorded, 1 µM tetrodotoxin (TTX) was included in the aCSF.

bioRxiv preprint doi: https://doi.org/10.1101/2023.12.21.572886; this version posted January 23, 2024. The copyright holder for this preprint (which was not certified by peer review) is the author/funder. This article is a US Government work. It is not subject to copyright under 17 USC 105 and is also made available for use under a CC0 license.

Asymmetric slices were obtained as previously described (Fischl & Weisz 2020). Briefly, the brainstem was carefully dissected from the skull to maintain a portion of the auditory nerve roots entering the cochlear nucleus, similar to methods used for thick slice preparations [27,28]. Then, a wedge-shaped section was acquired using a stage with an adjustable angle such that the lateral edge of one hemisphere was approximately 1-1.2 mm thick and contained the cochlear nucleus, and the opposite lateral edge was ~200 μm, creating a thickness of ~300-400 μm where MOC neurons were identified for patch-clamp experiments in the ventral nucleus of the trapezoid body (VNTB). An additional 300 μm slice was obtained rostral to the 'wedge slice' and used for additional experiments. For some experiments utilizing midline stimulation for evoked PSPs or for recordings of miniPSPs, symmetrical brain slices were prepared as previously described [18]. Sections were transferred to an incubation chamber and maintained at 35 ± 1°C for 30-60 min. Slices then cooled to room temperature until used for experiments within 4 hours of slicing. Wedge slices were used immediately after a short recovery incubation (30 min) to improve cell viability which tends to diminish more rapidly than typical symmetrical slices due to reduced aCSF solution penetration in the larger tissue volume.

Patch-clamp recordings

Sections were transferred to a recording chamber which was continuously perfused with aCSF at a rate of 5-10 mL/min. The bath temperature was held at 35 ± 1°C using an in-line heater (Warner) coupled to a temperature controller (Warner). The tissue was viewed using a Nikon Eclipse Ni-E microscope with an Apo LWD 25X/1.10 NA water-immersion objective attached to a Retiga Electro CCD camera (QImaging) operated using NIS Elements software (version 4.51.01). Epifluorescence illumination with red emission filters were used to locate MOC neurons in the VNTB for recordings. Targeted cells were then observed using DIC optics for patch-clamp recordings.

Pipettes for patch-clamp recordings were pulled from 1.5 mm borosilicate glass capillaries to resistances between 3-7 MOhm. For voltage-clamp experiments an internal solution containing (in mM) 76 Cs-methanesulfonate, 56 CsCl, 1 $MgCl_2$, 1 $CaCl_2$, 10 HEPES, 10 EGTA, 0.3 Na-GTP, 2 Mg-ATP, 5 Na_2-phosphocreatine, 5 QX-314, and 0.01 Alexa Fluor-488 hydrazide was

bioRxiv preprint doi: https://doi.org/10.1101/2023.12.21.572886; this version posted January 23, 2024. The copyright holder for this preprint (which was not certified by peer review) is the author/funder. This article is a US Government work. It is not subject to copyright under 17 USC 105 and is also made available for use under a CC0 license.

used. The pH was adjusted to 7.2 with CsOH. For current-clamp experiments, an internal solution containing (in mM) 125 K-gluconate, 5 KCl, 1 $MgCl_2$, 0.1 $CaCl_2$, 10 HEPES, 1 EGTA, 0.3 Na-GTP, 2 Mg-ATP, 1 Na_2-phosphocreatine, and 0.01 Alexa Fluor-488 hydrazide was used. The pH was adjusted 7.2 with 1N KOH. Liquid junction potentials were -6 mV, CsCl solution and -2 mV, KGlu solution and were not adjusted for. Electrophysiology recordings were performed using a HEKA EPC10 amplifier controlled using PatchMaster NEXT (version 1.1). The recordings were sampled at 50 kHz and filtered on-line at 10 kHz. Series resistance was compensated between 60-85%. Cells were voltage-clamped at -60 mV unless stated otherwise. In current-clamp, holding currents were injected to maintain the baseline membrane potential at -60 mV.

Stimulation of auditory nerve and ventral acoustic stria

Post-synaptic currents (PSC) and post-synaptic potentials (PSP) recorded in MOC neurons were evoked by electrical stimulation of axons using a bipolar tungsten electrode (WPI). For auditory nerve (AN) stimulation, the electrode was lowered onto the approximate center of the auditory nerve root diameter between the cut end of the nerve and its entry into the CN. For stimulation at the midline, the electrode was placed just lateral to the midline (contralateral hemisphere to the MOC neuron recording) near the ventral surface of the tissue onto fibers of the ventral acoustic stria. The stimulation was applied with an Iso-Flex Stimulus Isolation Unit (A.M.P.I.) and the intensity was adjusted to obtain consistent amplitude postsynaptic responses in MOC neurons (stimulation range 10-2000 μA). In two experiments using AN-stimulation, the stimulus intensity was increased until the PSC latencies jumped to a shorter value (see figure 3,4), indicating direct recruitment of CN axons and bypassing of auditory nerve synapses onto CN cells. To isolate inhibitory currents in voltage-clamp, the membrane potential was clamped at 0 mV, the approximate reversal potential for AMPA-mediated glutamatergic currents. Inhibitory inputs were blocked where indicated with bath application of strychnine (1 μM) and Gabazine (SR95531, 50 μM).

Miniature PSP recordings

For miniature PSP (mPSP) recordings, 1 μM tetrodotoxin (TTX, Alomone Labs) was included in the aCSF. Recordings were performed for several minutes to collect baseline (control) mPSPs.

bioRxiv preprint doi: https://doi.org/10.1101/2023.12.21.572886; this version posted January 23, 2024. The copyright holder for this preprint (which was not certified by peer review) is the author/funder. This article is a US Government work. It is not subject to copyright under 17 USC 105 and is also made available for use under a CC0 license.

Inhibitory inputs were blocked using bath application of strychnine (1 µM) and Gabazine (SR95531, 50 µM) and gap free recordings were again taken during pharmacological manipulation. mPSPs were detected using using MiniAnalysis software version 6.0.7 (Synaptosoft) using a threshold of 2 x RMS noise. PSCs were then accepted or rejected based on the characteristic PSC waveform. The decay time constants of PSCs were calculated in Mini Analysis software from individual events.

Calcium Imaging

Calcium imaging of activity in CN bushy cells was performed using the Atoh7/Math5 Cre mouse line crossed with a GCaMP6f mouse line (see above). Asymmetric wedge slices were prepared as described above (Experimental Model Details). aCSF used for recording calcium signals was modified to contain 2 mM $CaCl_2$. Epifluorescence illumination with green emission filters were used to locate bushy cell neurons in the anteroventral cochlear nucleus (AVCN). The AVCN was targeted to increase the potential for imaging of globular bushy cells (GBCs) which project to the contralateral MNTB. Calcium signals were imaged using a Nikon Eclipse Ni-E microscope with an Apo LWD 25X/1.10 NA water-immersion objective in 2-photon excitation mode at 920 nm (Mai Tai HP, Spectra-Physics). A single focal plane was imaged for data collection. Imaging was performed at 3 Hz for 20 seconds using the resonant scanning galvos (Nikon Elements software version 4.51.01). Protocols consisted of 5 seconds of baseline data collection followed by auditory nerve stimulation in 3 bouts (each bout is 20 pulses at 100 Hz), at 5 seconds intervals, followed by an additional 5 seconds after the third stimulation bout, then at least 30 seconds without imaging or stimulation. Control data was acquired for several minutes before glutamate receptor blockers were applied. In a subset of experiments, CNQX (5 µM) was applied alone to block ionotropic glutamate receptors. In remaining calcium imaging experiments, APV (50 µM) was also added to additionally block NMDA receptors. Acquisition protocols were repeated during blocker application. Blockers were applied for ~10 minutes before washout. After washout, protocols were repeated to assess recovery of control conditions.

Computational MOC neuron model

A model of a single MOC neuron was constructed using NEURON v8 [124] and Python 3. The neuron topology was generated from a published MOC neuron morphology (Figure 9 from,

bioRxiv preprint doi: https://doi.org/10.1101/2023.12.21.572886; this version posted January 23, 2024. The copyright holder for this preprint (which was not certified by peer review) is the author/funder. This article is a US Government work. It is not subject to copyright under 17 USC 105 and is also made available for use under a CC0 license.

[47]). The number of segments was empirically determined to be 86, inserted using the nseg function, and compartments were organized into larger morphological groups including the soma (length 33.6 µm, diameter 6.1 µm), axon (length 180.0 µm, diameter 1 µm), and dendrites. The three primary dendrites projected from the soma (lateral: length 12.8 µm, diameter 1 µm), dorsal (length 10.1 µm, diameter 1 µm), and medial (length 7.3 µm, diameter 1 µm). Each of these three primary dendrites branched further into 8 to 46 dendrites with lengths from 1.5 to 31.4 µm, and diameters from 1-2 µm. This detailed neuron topology allowed specific control of physiological properties. The uniform axial resistance was 210 ohm-cm, and membrane capacitance was 1 µF/cm^2. Channels were inserted into the membrane (Table 1) to recapitulate our experimental results. HCN channel reversal potential was set to -38 mV. The model was run at 35 C.

Post-synaptic potentials (PSP) were simulated at the soma to replicate recorded values. The model MOC neuron responded to synaptic inputs based on recorded mini-EPSPs (mEPSP) and mini-IPSPs (mIPSP) with output amplitudes and waveforms closely matching recorded values (mean±SD; mEPSP: amplitude: 0.86±0.23 mV, time constant of decay: 8.65±1.43 ms, n=6 neurons; mIPSP: amplitude: -1.57±1.41 mV, time constant of decay: 18.1±9.68 ms, n=7 neurons; model MOC mEPSP: amplitude: 0.90 mV, time constant of decay: 8.21 ms; model MOC mIPSP: amplitude: -1.56 mV, time constant of decay: 14.4 ms.

Next, synaptic potentials were simulated within the model MOC neuron to mimic PSPs evoked from MdL-stimulation experiments both in control conditions, and with pharmacological blockade of inhibitory synaptic inputs (MOC recording control evoked-PSP: amplitude: 1.88±0.95 mV, time constant of decay: 10.7±4.00 ms, n=9 neurons; inhibition blocked evoked-PSP (EPSP only): amplitude: 2.20±1.17 mV, time constant of decay: 14.1±7.50 ms, n=9 neurons; model MOC control evoked-PSP: amplitude: 1.64 mV, time constant of decay: 14.8 ms; model MOC inhibition blocked evoked-PSP (EPSP only): amplitude: 1.91 mV, time constant of decay: 16.4 ms. To achieve these output parameters, model MOC neuron input values were as follows: MOC model EPSP input parameters: rise time = 0.2 ms, time constant of decay = 6 ms, synaptic weight = 0.0015, reversal potential = 0 mV, resulting amplitude = 8.506 mV). The inhibitory PSP was designed so that simulation of EPSPs and IPSPs in the model replicated control MOC neuron current-clamp recordings of evoked PSPs. The model MOC IPSP output parameters were amplitude: -0.269 mV, time constant of decay: 14.8 ms. To achieve

bioRxiv preprint doi: https://doi.org/10.1101/2023.12.21.572886; this version posted January 23, 2024. The copyright holder for this preprint (which was not certified by peer review) is the author/funder. This article is a US Government work. It is not subject to copyright under 17 USC 105 and is also made available for use under a CC0 license.

these values, the model MOC IPSP input parameters were as follows: rise time = 2.75 ms, time constant of decay = 3.64, synaptic weight 0.00032, reversal potential = -90 mV, resulting amplitude = -0.536 mV).

PSPs with excitatory and inhibitory components were simulated with onset timing that systematically changed in 1 ms increments from excitation-inhibition latencies (E-I latency) from -10 (IPSPs precede EPSPs by 10 ms) to +10 (EPSPs precede IPSPs by 10 ms). E-I latencies were also simulated within the model to replicate recorded PSC latencies (Results text). PSPs were then simulated within the model in trains of 20 pulses at 100 Hz, using either the excitation-only PSP, or the combined PSPs with E-I latencies that systematically varied between -10 and +10, in 1 ms intervals.

The model will be available at Model dB (identifier # TBD at acceptance)

**Channels**	**Location (segments)**	**Conductance**
High Threshold K^+ (h.HT)	All	0.02 S/cm^2
Low Threshold K^+ (h.LT)	All	12 mS/cm^2
Low Threshold K^+ (h.kbl_LT)	All	0.6 ms^-1
H-H type Na^+ (h.na)	All	8 nS/cm^2
HCN (h.Ih_400t8)	All	12 $\mu S/cm^2$
High Threshold K^+ (h.HT)	Axon	0.32 mS/cm^2
Low Threshold K^+ (h.LT)	Axon	6 cS/cm^2
H-H type Na^+ (h.na)	Axon	9 kS/cm^2

Methods Table 1. Biophysical properties of a modeled MOC neuron compared to measured values recorded from MOC neurons.

QUANTIFICATION AND STATISTICAL ANALYSIS

Statistics for PSCs

Analysis of synaptic inputs to MOC neurons required classification of evoked PSCs as excitatory or inhibitory. Clampfit software was used to detect individual PSCs for analysis. Latency to PSC onset, rise time, latency to PSC peak, amplitude and decay time constant (tau decay) were measured for PSCs recorded at both –60 mV and 0 mV holding potential. A single exponential

bioRxiv preprint doi: https://doi.org/10.1101/2023.12.21.572886; this version posted January 23, 2024. The copyright holder for this preprint (which was not certified by peer review) is the author/funder. This article is a US Government work. It is not subject to copyright under 17 USC 105 and is also made available for use under a CC0 license.

function fit was used to calculate the time constant of decay (τ). These data were then used for individual cell clustering analysis (below).

Individual cell clustering analysis

Clustering analysis was performed using PSC metrics in order to sort PSCs into statistically defined clusters. Clustering was performed with PSCs collected at both –60 mV and 0 mV holding potential, when available. For each cell's PSCs, values for onset latency, rise time, amplitude and decay time constant collected from Clampfit were imported into R. Function libraries utilized for clustering included {parameters}, {factoextra} and {NbClust}. The appropriate number of clusters was determined using the gap statistic method (Tibshirani et al. 2001). This method was chosen because of its ability to select "one" as the optimal number of clusters where appropriate. The "clusGap" function was used with kmax=10 (max# of clusters), nstart=25 (# of random start centers) and B=500 (bootstrapping). Once the appropriate number of clusters was determined, a k-means cluster analysis was performed in R using the "kmeans" function to sort the PSCs into clusters (with centers=output # from gap statistic analysis and nstart=25). Once the PSCs were assigned a cluster number, statistical analyses were performed on each cluster. For PSCs collected with midline stimulation where data was acquired at 0 mV holding potential, a cluster was deemed inhibitory if it was present at both -60 mV and 0 mV. A cluster was categorized as excitatory if PSCs from the cluster were-only present at -60 mV. This was a robust categorization, with only two out of eleven cells having a PSC mis-identified in the cluster, in both cells a single excitatory PSC was classified into an inhibitory cluster (2 out of 654 mis-identified PSCs). PSCs acquired with auditory nerve (AN) stimulation were analyzed via the same cluster analysis. AN stimulation PSCs were categorized as excitatory or inhibitory using a machine learning algorithm (see below). Statistical comparisons between excitatory and inhibitory PSC clusters were then made across the population.

Machine Learning Algorithm to classify post-synaptic currents (PSCs)

A RandomForest machine learning algorithm was utilized to classify recorded PSCs as excitatory or inhibitory based on the variables of PSC rise time, time constant of decay, amplitude, probability of occurrence within a cluster, onset jitter within a cluster, peak jitter within a cluster, and animal age. All values were continuous except for animal age, which was

bioRxiv preprint doi: https://doi.org/10.1101/2023.12.21.572886; this version posted January 23, 2024. The copyright holder for this preprint (which was not certified by peer review) is the author/funder. This article is a US Government work. It is not subject to copyright under 17 USC 105 and is also made available for use under a CC0 license.

treated as a categorical variable. The model included 952 PSCs recorded in the ML-stimulation configuration at a holding potential of -60 mV, from 22 MOC neurons. These PSCs had been previously classified as excitatory or inhibitory based on recordings from the same neurons at a holding potential of 0 mV and the clustering analysis described below. After training the RandomForest algorithm on this data, the model classification accuracy reached 99.89%, with out-of-bag (OOB) error stabilization at 150 'trees'. Re-running the algorithm on the training dataset determined that it was able to distinguish excitatory and inhibitory events perfectly with an area under the curve (AUC) of 1, indicating excellent ability of the model to distinguish excitatory vs inhibitory PSCs. The model was then used to classify the individual PSCs in the AN stimulation dataset recorded at -60 mV as excitatory or inhibitory. First, the RandomForest algorithm determined the probability that each of the 344 PSCs was excitatory or inhibitory. PSCs were given the classification that had the highest probability (>0.5) by the algorithm. The algorithm gave slightly higher classification probabilities for excitatory (0.81±0.14, n=181) compared to inhibitory (0.75±0.12, n=163) PSCs. AN stimulation PSCs were grouped into 28 clusters determined above through cluster analysis. Three clusters contained both excitatory and inhibitory PSCs. Two cells had a majority of one classification (7 of 10 excitatory and 19 of 21 excitatory and one cell was approximately split 6 of 11 excitatory). For further analyses, we separated these three mixed clusters into an excitatory and inhibitory cluster each to yield a total of 31 AN-stimulation clusters.

Calcium Imaging

After acquisition of fluorescent signals in cochlear nucleus bushy cells in Atoh7/Math5 Cre; GCaMP6f wedge slices (see above), fluorescence changes in response to electrical stimulation of axons were measured to determine the effect of synaptic stimulation of bushy cells with and without blockers of post-synaptic receptors. Polygonal ROIs were drawn by hand around neurons and any major processes that could be resolved (Elements software version 4.51.01), and average intensity values for ROIs were calculated for each frame. Maximum fluorescence elicited from AN stimulation within a protocol was compared to baseline average (F) of each ROI. Fluorescence change (ΔF) and relative fluorescence change (ΔF/F) was calculated using Excel. Heat maps were constructed from the intensity value output of a given frame from the Elements software using custom MATLAB scripts. Baseline values were calculated as the

bioRxiv preprint doi: https://doi.org/10.1101/2023.12.21.572886; this version posted January 23, 2024. The copyright holder for this preprint (which was not certified by peer review) is the author/funder. This article is a US Government work. It is not subject to copyright under 17 USC 105 and is also made available for use under a CC0 license.

average pixel intensity of the first 15 frames (~5 seconds) before axon stimulation. Cells were considered active if the average fluorescence of an ROI reached two standard deviations (SD) above the baseline average in at least 2 of the 3 stimulations during a protocol. Active cells were then used to compare the ΔF/F between control and glutamate block conditions.

Data analysis and statistics

Statistical analyses were performed in Origin (v2021 and v2022). Normality tests were performed on data sets using the Shapiro-Wilk test. The majority of data sets were non-normally distributed so non-parametric testing was employed. The Mann-Whitney U test was used for testing between two independent groups. A One-Sample Wilcoxon Signed Rank Test determined whether E-I latency difference values were significantly different from zero for MdL- and AN-stimulation PSCs. Action potential metrics collected at different stimulus rates (Figure 6) were compared in the control condition using Friedman's ANOVA. Post-hoc Dunn's test was used to test significance between stimulus rates. Action potential metrics were also compared between control and inhibition block conditions using paired Wilcoxon Signed Rank Test. Calcium imaging data were analyzed using Kruskal-Wallis ANOVA where post-hoc Dunn's test was used to test whether control data sets between the CNQX and CNQX+APV conditions were significantly different from each other. Population data are summarized in box plots with the box representing the 1st and 3rd quartiles, the line representing the median, the square representing the mean and the error bars representing the 10th and 90th percentiles. Figures were prepared in Origin and Adobe Illustrator.

bioRxiv preprint doi: https://doi.org/10.1101/2023.12.21.572886; this version posted January 23, 2024. The copyright holder for this preprint (which was not certified by peer review) is the author/funder. This article is a US Government work. It is not subject to copyright under 17 USC 105 and is also made available for use under a CC0 license.

Supplementary Tables

	MdL-EPSC	MdL-EPSC n (clusters / cells)	MdL-IPSC	MdL-IPSC n (clusters / cells)	p-value (Mann-Whitney U Test)
Amplitude, -60 mV (pA)	66.95±3.95	25/17	55.39±3.10	21/15	0.69
Rise Time (ms)	0.63±0.19	25/17	0.86±0.15	21/15	8.02E-04
Time constant of decay (τ, ms)	2.23±0.52	23/17	3.51±0.93	21/15	4.95E-07
Latency (ms)	1.92±0.37	18/18	4.47±0.93	15/15	4.79E-06

Table S1. Parameters of MdL-stimulation-evoked PSCs from Figure 1D, for all clusters.

	ΔF/F	p-value	% suppression from control
control (106)	1.176±0.075		
CNQX	1.059±0.018	1.04E-20	67.67±12.43
CNQX wash	1.150±0.073	2.74E-13	
control (121)	1.160±0.071		
CNQX + APV	1.039±0.009	1.14E-34	74.51±11.31
CNQX + APV wash	1.125±0.055	2.54E-24	

Table S2. Top: Fluorescence changes in Atoh7/Math5Cre; GCaMP6f expressing bushy cells in control conditions, with CNQX, and wash of CNQX. Bottom: Fluorescence changes in control, with CNQX + APV, and wash of CNQX + APV. Differences between conditions tested with Friedman's ANOVA followed by post-hoc Dunn's Test. % suppression from control: (control DF/F-block DF/F) / (control DF/F-1)*100%. Number of cells for each set of experiments in parentheses.

	AN-EPSC	AN-EPSC n (clusters / cells)	AN-IPSC	AN-IPSC n (clusters / cells)	p-value (Mann-Whitney U Test)
Amplitude -60 mV (pA)	60.6±39.0	13/8	25.6±11.1	17/10	0.14
Rise Time (ms)	0.86±0.15	13/8	0.92±0.31	17/10	0.35
Time constant of decay (τ, ms)	2.80±1.65	11/8	3.59±1.50	16/10	0.67
Latency (ms)	5.83±1.22	13/8	6.71±0.87	17/10	0.04

Table S3. Parameters of AN-stimulation evoked PSCs from Figure 4E, for all clusters.

bioRxiv preprint doi: https://doi.org/10.1101/2023.12.21.572886; this version posted January 23, 2024. The copyright holder for this preprint (which was not certified by peer review) is the author/funder. This article is a US Government work. It is not subject to copyright under 17 USC 105 and is also made available for use under a CC0 license.

	MdL-EPSC (18)	MdL-IPSC (15)	AN-EPSC (8)	AN-IPSC (11)
Latency (ms)	1.92±0.37	4.47±0.93	5.22±0.98	6.19±0.77
Onset Jitter (ms)	0.25±0.13	0.50±0.17	0.37±0.13	0.56±0.11
Probability	0.80±0.19	0.45±0.15	0.24±0.08	0.14±0.06

Table S4. Parameters of the first cluster of PSCs evoked from MdL- and AN-stimulation from Figure 5. Numbers in parentheses indicate number of cells.

Rate	Treatment (n)	AP Prob	AP Rate (Hz)	# stimulations to first AP	Latency to first AP
10 Hz	Control (7)	0	0	>20	>2000
10 Hz	Block (7)	0	0	>20	>2000
10 Hz	Wash (5)	0	0	>20	>2000
50 Hz	Control (7)	0.005±0.005	0.25±0.25	19.0±1.0	379.2±20.9
50 Hz	Block (7)	0.053±0.053	2.63±2.63	7.80±4.40	144.4±86.87
50 Hz	Wash (5)	0	0	>20.0	>400
100 Hz	Control (7)	0.035±0.015	3.49±1.51	14.15±4.30	139±44.6 #
100 Hz	Block (7)	0.078±0.036 *	7.78±3.75 *	5.40±2.76 *	49.8±29.1 *
100 Hz	Wash (5)	0.051±0.03	5.08±3.00	9.50±5.13	126±56.7
200 Hz	Control (7)	0.045±0.040 #	9.00±8.00 #	14.2±4.20 #	69.3±22.3 #
200 Hz	Block (7)	0.075±0.047 *	15.0±9.40 *	6.00±2.00 *	26.3±12.0 *
200 Hz	Wash (5)	0.013±0.013	2.67±2.67	16.6±3.50	75.7±19.5

Table S5. Measurements of AP probability, rate, the number of stimulations to first AP, and latency to first AP in 5-8 MOC neuron recordings in response to MdL-stimulation at the frequency indicated, from Figure 6C. Measurements are presented in control, during pharmacological blockade of post-synaptic inhibitory receptors ("block"), and wash conditions, as median±MAD. Numbers in parentheses indicate n. # indicates significantly different from control 10 Hz stimulation (Friedman ANOVA with post-hoc Dunn's test). * indicates significantly different from control within stimulus frequency comparison (P<0.05, Paired Wilcoxon Signed-Rank Test).
